# Surgical resection for clinical stage I high-grade neuroendocrine carcinoma of the lung

**DOI:** 10.1186/s12957-018-1337-2

**Published:** 2018-02-17

**Authors:** Eisuke Mochizuki, Shun Matsuura, Kyohei Oishi, Koichi Miyashita, Koshiro Ichijyo, Syunya Furukawa, Miyuki Nagaoka, Shinichiro Mikura, Masaru Tsukui, Naoki Koshimizu, Shogo Sakurai, Kazuhiro Asada, Toshihiro Shirai

**Affiliations:** 1 Department of Respiratory Medicine, Fujieda Municipal Hospital, 4-1-11 Surugadai, Fujieda, Shizuoka 426-8677 Japan; 2 Department of Respiratory Medicine, Shizuoka General Hospital, 4-27-1 Kita-Ando, Aoi, Shizuoka, 420-8527 Japan

**Keywords:** Lung cancer, High-grade neuroendocrine carcinoma, Surgery

## Abstract

**Background:**

There are few reports about the factor influencing the prognosis of high-grade neuroendocrine carcinoma. In this study, we evaluated surgical outcome of clinical stage I high-grade neuroendocrine carcinoma.

**Methods:**

Patients who underwent curative surgery for high-grade neuroendocrine tumors of the lung in clinical stage I were included in this study. We retrospectively analyzed 27 consecutive patients. The aim of this study was to clarify the clinical course of the disease after surgery and what factors influence the prognosis.

**Results:**

Twenty-two patients have small cell carcinoma, and 5 patients have large cell neuroendocrine carcinoma. Patients who could undergo surgery within 60 days after the first visit (*p* < 0.01) and undergo lobectomy (*p* < 0.01) and whose pro-gastrin-releasing peptide ≦ 72 pg/ml (*p* = 0.04) performed good prognosis after surgery. In multivariate analysis, surgery within 60 days and operative procedure were independent factors associated with OS.

**Conclusion:**

Surgical resection for clinical stage I high-grade neuroendocrine carcinoma of the lung should be performed as early as possible, and better outcome can be obtained with lobectomy than partial resection.

## Background

Patients with lung cancer have better outcome, when they underwent surgical resection in early stages. We can perform surgical resection for non-small cell carcinoma patients; however, for high-grade neuroendocrine carcinomas (small cell carcinoma and large cell neuroendocrine carcinoma) as they are rapidly progressive, most cases are inoperable when they are diagnosed. There are few reports about the prognosis and the course of the patients with high-grade neuroendocrine carcinomas in early stage after surgery. The aim of this retrospective study was to clarify the clinical course of the disease after surgery and what factors influence the prognosis.

## Methods

This retrospective study was conducted at Fujieda Municipal Hospital and Shizuoka General Hospital in Japan. We collected the data of 27 patients who underwent curative surgery for high-grade neuroendocrine tumors of the lung in clinical stage I (22 small cell carcinoma and 5 large neuroendocrine carcinoma) from January 2005 through January 2015.

Before surgery, all patients underwent a computed tomography (CT) scan of the chest and abdomen and magnetic resonance imaging of brain as well as positron-emission tomography (PET) whenever possible. In PET, lymph nodes with a standardized uptake value (SUV) of 2.5 or greater were considered positive. For patients who did not undergo PET, lymph nodes which short axis were greater than 1.0 cm in CT scan were considered positive. Staging was based on the seventh edition of the Union for International Cancer protocol (UICC) TNM staging system. We defined the date of the first visit to our hospital or the date of chest X-ray performed by family doctor as the initial date. The preoperative interval was defined as the time between the initial date and the operation. Since pathological information of the lymph nodes of the patients who underwent partial lung resection could not be assessed, in such cases, we regarded clinically N0 as pathologically N0. No patient underwent induction chemotherapy nor prophylactic cranial irradiation. All patients were measured pro-gastrin-releasing peptide (Pro-GRP) before operation. Adjuvant chemotherapy was inducted after surgery if the patients did not refuse. Cisplatin (or carboplatin) + etoposide (or irinotecan) was used to them.

Comparisons between groups were made by Mann-Whitney *U* test. The chi-square or Fisher’s exact test was used to test significance in the group differences with respect to the percentage of patients in the various categories. The Kaplan-Meier method with the log-rank test was used to represent the unadjusted factor’s associated survival. Variables were included in the model if they were statistically significant in affecting survival in univariate analysis. Median values are shown with the 95% confidence interval (CI). All statistical analyses were performed with EZR (Saitama Medical Center, Jichi Medical University, Saitama, Japan), which is a graphical user interface for R (The R Foundation for Statistical Computing, Vienna, Austria). More precisely, it is a modified version of R commander designed to add statistical functions frequently used in biostatistics [1].

A *p* value of < 0.05 was considered significant, and all tests were two sided.

The protocol was approved by the local ethics committee of Fujieda Municipal Hospital and performed in accordance with the ethical standards.

## Results

All patients were male, with a median age of 73 years (range, 59 to 81). Twenty-one patients had a history of cigarette smoking, and median pack-years were 46 (range, 0 to 104). Median maximum diameter of tumor was 22 mm (range, 11 to 48).

Nodules were found in 20 patients during routine medical checkups, in 6 patients during chest X-rays or CT scans for other disease checkups, and in 1 patient due to cough.

Twenty-two patients underwent PET-CT before surgery, and all patients received complete preoperative staging via enhanced CT scans from the neck to the pelvis as well as brain MRI.

The numbers of the patients in clinical stages of IA and IB were 21 and 6, respectively.

Of the 21 patients in clinical stage IA, the pathological stage was IA, 15 patients; IB, 4 patients; IIA, 1 patient; and IIIA, 1 patient. Of the 6 patients in clinical stage IB, the pathological stage was IA, 1 patient; IB, 2 patients; IIA, 1 patient; IIB, 1 patient; and IIIA, 1 patient. Twenty-one patients underwent lobectomy with hilar and mediastinal lymph node dissection, while 6 patients underwent partial resection without lymph node dissection. No death was reported in the perioperative period. Of the 27 patients, 22 received postoperative adjuvant chemotherapy (Table 1).

**Table 1:** Characteristics of patients

Characteristic	Patients (*n* = 27)
Age	73 (59–81)
Sex	
Male	27
Female	0
Pathology	
Small	22
LCNEC	5
Smoking history (pack-years)	46 (0–104)
Maximum tumor diameter (mm)	22 (11–48)
Clinical stage	
IA	20
	Small cell carcinoma 16
	LCNEC 4
IB	7
	Small cell carcinoma 6 LCNEC 1
Pathological stage	
IA	15
	Small cell carcinoma 14
	LCNEC 1
IB	7
	Small cell carcinoma 7
	LCNEC 0
IIA	2
	Small cell carcinoma 1
	LCNEC 1
IIB	1
	Small cell carcinoma 0
	LCNEC 1
IIIA	2
	Small cell carcinoma 1
	LCNEC 1
Preoperative Pro-GRP (pg/ml)	43.4 (21.6–205)
Operative procedure	
Lobectomy	21
Partial resection	6
Adjuvant chemotherapy	22
Interval from first visit operation (day)	51 (27–159)
Diagnosis before operation	3

Data are expressed as medians (ranges) or number (%)

**Fig. 1:**
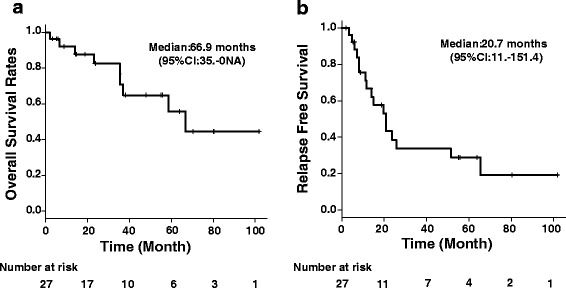
Kaplan-Meier curve for overall survival and relapse-free survival of the patients. **a** Median overall survival. **b** Median relapse-free survival

Median relapse-free survival (RFS) and overall survival (OS) were 20.7 months (95% CI 11.1–51.4) and 66.9 months (95% CI 35.0–NA), respectively (Fig. 1).

**Fig. 2:**
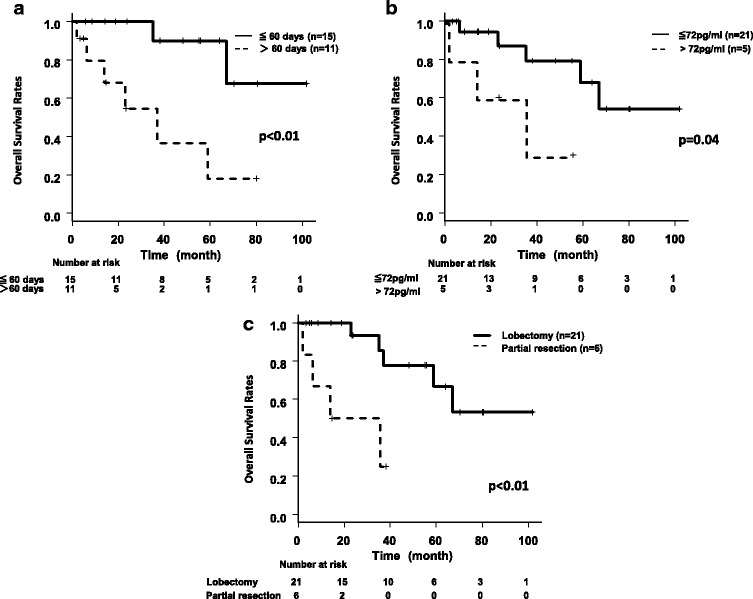
Kaplan-Meier curves for overall survival of the patients according to the clinical factors. **a** Overall survival of high-grade neuroendocrine tumors divided by interval from the first visit operation. **b** Overall survival of high-grade neuroendocrine tumors divided by Pro-GRP. **c** Overall survival of high-grade neuroendocrine tumors divided by operative procedure

**Fig. 3:**
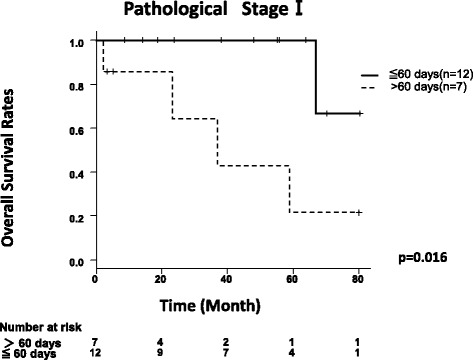
Kaplan-Meier curves for overall survival rates of the pathological stage I patients. There were 12 patients in pathological stage I with an interval < 60 days. All 12 patients underwent lobectomy. There were 7 patients in pathological stage I with an interval > 60 days. Six of these patients underwent lobectomy with mediastinal and hilar nodal dissection, and 1 patient received partial resection

Fifteen patients who underwent surgery within 60 days after the first visit demonstrated a better prognosis regarding OS (*p* < 0.01) than those underwent on after 60 days (one patient missed data) (Fig. 2a). This result is also true in pathological stage I patients; OS of the patients operated within 60 days from initial visit was better than that of the patients operated at over 60 days (Fig. 3).

Sixteen patients whose Pro-GRP before operation were below 72 pg/ml demonstrated a better prognosis regarding OS than those whose Pro-GRP were over 72 pg/ml (*p* = 0.04) (Fig. 2b). Twenty-one patients who underwent lobectomy with hilar and mediastinal lymph node dissection also demonstrated better OS than patients who underwent partial resection (Fig. 2c). The diagnosis, smoking history (pack-years), the size of the tumor, adjuvant chemotherapy, clinical stage (IA or IB), pathological stage (IA or IB), and age did not influence OS.

With a Cox proportional hazard analysis, four clinical factors were examined: age, preoperative Pro-GRP, interval from the first visit operation, and operative procedure. In multivariate analysis, the first visit operation and operative procedure were independent factors associated with OS (Table 2).

**Table 2:** The four clinical factors that are examinedᅟ

Factor	Hazard ratio	95% CI	*p* value
Age	0.992	0.841–1.170	0.924
Preoperative Pro-GRP	2.108	0.287–15.510	0.464
Interval from the first visit to operation	6.393	1.042–39.220	0.045
Operative procedure	10.06	1.141–88.75	0.037

The statistical significance was tested by multivariate Cox hazards models

*CI* confidence interval

Among the 27 patients, 17 relapsed and 8 did not. We could not track the relapse data for 2 patients who moved to another hospital.

We analyzed the data for the 25 patients we could follow. Patients who underwent surgery after 60 days showed a significant difference in distant relapse (*p* = 0.02).

## Discussion

Long-term survival could be obtained in small cell carcinoma in early stage if the resection and adjuvant chemotherapy were performed appropriately. Niiranen et al. reported good prognosis could be obtained with combination of surgery and adjuvant chemotherapy [2], whereas Namikawa et al. reported the 5-year survival rate of patients of small cell carcinoma with stage I was 75% [3]. It is clear that the operation of lung cancer should be performed as soon as possible when it is operable, especially in high-grade neuroendocrine carcinomas, as they grow rapidly and can become inoperable within several months. Large cell neuroendocrine carcinoma represents 1 to 2% of malignant pulmonary neoplasms [4]; however, it is unclear that by what time we should operate after they were recognized. This study was designed to evaluate what factor affected OS of high-grade neuroendocrine carcinoma operation.

A high titer for preoperative serum Pro-GRP has been associated with poor OS (*p* = 0.04). Pro-GRP is a member of the bombesin family of peptides shown to have mitogenic activity in small cell lung carcinoma and is produced by small cell lung carcinoma in an autocrine fashion. Sunaga et al. reported serum Pro-GRP is a useful marker for treatment monitoring and survival in small cell lung cancer [5]. In non-small cell lung carcinoma, Okada et al. analyzed 1000 patients with clinical stage I non-small cell lung carcinoma and found that preoperative serum carcinoembryonic antigen (CEA) levels were an independent prognostic factor [6] and Kinoshita et al. also reported high-grade serum CEA levels, lymphatic permeation, and vascular invasion were independent prognostic factors [7]. Although there was no report of preoperative prognostic serum tumor marker of high-grade neuroendocrine carcinoma, from the result of our study, preoperative Pro-GRP could be the prognostic factor after operation.

Moreover, lobectomy with hilar and mediastinal lymph node dissection demonstrated better OS than patients who underwent partial resection. Ginsberg et al. performed a prospective randomized trial of lobectomy versus limited resection for T1 N0 non-small cell lung cancer. In their study, higher death rate and locoregional recurrence rate were reported with limited resection [8]. Furthermore, Allen et al. and Darling et al. prospectively evaluated the prognostic significance of lymph node dissection in lung cancer [9, 10]. On the basis of the combination of these results, it is widely accepted that lobectomy with hilar and mediastinal lymph node dissection/sampling is the present-day gold standard for the lung cancer. On the other hand, there are a few retrospective study of surgical resection for small cell carcinoma of the lung. Weksler et al. reported about surgical resection for stage I and II small cell carcinoma of the lung. In their study, patients who underwent lobectomy or pneumonectomy had better OS compared with patients who underwent a wedge resection [11]. In our study, partial resection was mainly adopted whose pulmonary function was not competent, but lobectomy with hilar and mediastinal lymph node dissection demonstrated better OS than patients who underwent partial resection. This is also true of other studies.

Besides, in clinical stage I patients, OS of the patients operated within 60 days from the initial visit was better than that of the patients operated at over 60 days. And also, in pathological stage I patients, OS of the patients operated within 60 days from the initial visit was better than that of the patients operated at over 60 days (Fig. 3). Although there was no significant pathological difference between patients operated within 60 days and patients operated at over 60 days, OS was different. This was a remarkable result, which showed that poor OS was not derived from pathological progression. From our result, we can speculate tiny metastasis, beyond thorax, could start to appear as time went by.

We also analyzed the relapse data for the patients. Of the 27 patients, 17 relapsed, 8 did not, and we could not determine the relapse status for the other 2 because they move to another hospital.

For the 17 patients who relapsed, disease relapse was locoregional for 10 patients and distant for 7 patients. Patients who underwent operation over 60 days showed a significant difference in distant relapse (*p* = 0.02). Therefore, we are certain that tiny metastases, beyond the thorax, began to appear over time.

In a study by Buccheri et al., delays in time between the presentation of first symptoms and consultation with a specialist were examined in 1277 patients with stage I–IV non-small cell carcinoma lung cancer [12]. They found a small but statistically significant decrease in survival in patients with delays greater than 2 months compared with those who waited less than 2 months. Thus, we defined surgical delay as over 60 days. Though the difference of biological behavior between high-grade neuroendocrine carcinoma and non-small lung cancer is different, similar result was obtained. To our knowledge, this is the first report to study surgical wait time on high-grade neuroendocrine carcinoma.

There are some limitations to this study. This study is retrospective and relatively small size and does not take account of patient comorbidities or quality of life. We could perform only retrospective analysis about operation interval on ethical reason. And next, we could not assess mediastinal lymph node precisely. There were potential inappropriate inclusion of patients who have had metastasis of mediastinal lymph node that were not identified on CT or PET scans.

To our knowledge, the present study is the only one to assess the effect of time to the operation of high-grade neuroendocrine carcinoma. Many factors were responsible for the delays between the first clinical presentation and definite surgical therapy such as patient refusal of operation, rescheduling operation because of pneumonia, and re-biopsy because of negative results; there were many operations awaiting to perform within 60 days. The cooperation among doctors (family doctor, internal medicine doctor, and surgeon) is important especially for rapidly progressive tumor to avoid delay of treatment. Although further accumulation of study is desirable to obtain more information, early operation is preferable especially in high-grade neuroendocrine carcinoma.

## Conclusions

Surgical resection for high-grade neuroendocrine carcinoma with stage I should be performed within 60 days after the first visit.

## Abbreviations

CEA: Carcinoembryonic antigen
CI: Confidence interval
CT: Computed tomography
OS: Overall survival
PET: Positron-emission tomography
Pro-GRP: Pro-gastrin-releasing peptide
RFS: Relapse-free survival
SUV: Standardized uptake value
UICC: Union for International Cancer protocol
